# Early immune responses and prognostic factors in children with COVID-19: a single-center retrospective analysis

**DOI:** 10.1186/s12887-021-02561-y

**Published:** 2021-04-17

**Authors:** Wenjie Lu, Li Yang, Xiong Li, Ming Sun, Aiping Zhang, Shanshan Qi, Zhi Chen, Lannan Zhang, Jianxin Li, Hao Xiong

**Affiliations:** 1 Department of Hematology, Wuhan Children’s Hospital (Wuhan Medical Care Center for Women and Children), Tongji College, Huazhong University of Science and Technology, Wuhan, Hubei China; 2 Department of Surgery, University of Michigan School of Medicine, Ann Arbor, MI USA; 3 Center of Excellence for Cancer Immunology and Immunotherapy, University of Michigan Rogel Cancer Center, University of Michigan School of Medicine, Ann Arbor, MI USA

**Keywords:** SARS-CoV-2, Children, Severe case, Lymphocyte subset, Inflammatory factor

## Abstract

**Background:**

Early diagnostic indicators and the identification of possible progression to severe or critical COVID-19 in children are unknown. To investigate the immune characteristics of early SARS-CoV-2 infection in children and possible key prognostic factors for early identification of critical COVID-19, a retrospective study including 121 children with COVID-19 was conducted. Peripheral blood lymphocyte subset counts, T cell-derived cytokine concentrations, inflammatory factor concentrations, and routine blood counts were analyzed statistically at the initial presentation.

**Results:**

The T lymphocyte subset and natural killer cell counts decreased with increasing disease severity. Group III (critical cases) had a higher Th/Tc ratio than groups I and II (common and severe cases); group I had a higher B cell count than groups II and III. IL-6, IL-10, IFN-γ, SAA, and procalcitonin levels increased with increasing disease severity. Hemoglobin concentration, and RBC and eosinophil counts decreased with increasing disease severity. Groups II and III had significantly lower lymphocyte counts than group I. T, Th, Tc, IL-6, IL-10, RBC, and hemoglobin had relatively high contribution and area under the curve values.

**Conclusions:**

Decreased T, Th, Tc, RBC, hemoglobin and increased IL-6 and IL-10 in early SARS-CoV-2 infection in children are valuable indices for early diagnosis of severe disease. The significantly reduced Th and Tc cells and significantly increased IL-6, IL-10, ferritin, procalcitonin, and SAA at this stage in children with critical COVID-19 may be closely associated with the systemic cytokine storm caused by immune dysregulation.

## Introduction

The coronavirus disease 2019 (COVID-19) outbreak in Wuhan, Hubei Province, China, in late December 2019 has been controlled by effective measures. However, the epidemic outside China has not been controlled effectively. Compared with adult patients, most children are infected with severe acute respiratory syndrome coronavirus 2 (SARS-CoV-2) through family clusters, with relatively mild clinical symptoms and a good prognosis [1]. Several studies have suggested that, in adults infected with SARS-CoV-2, the progression to severe cases is clearly associated with cytokine storm or cytokine storm syndrome, which is life-threatening and clinically characterized by systemic inflammation, hyperferritinemia, hemodynamic instability and multiple organ failure, and it is an important factor contributing to the life-threatening clinical manifestations of H7N9, H5N1, and SARS [2–4]. To date, there has been no report on the indications for early diagnosis and identification of possible progression to severe or critical cases in children with SARS-CoV-2 infection.

We retrospectively collected the clinical data of 121 children with confirmed and highly suspected SARS-CoV-2 infection who had been admitted to Wuhan Children’s Hospital from January 25, 2020, to February 20, 2020, and analyzed the differences in the laboratory indices of peripheral blood from children with early mild and severe SARS-CoV-2 infection. We investigated the differences in the immune and stress responses in children with different disease severity in the early stage of SARS-CoV-2 infection, and aimed to provide a laboratory basis for early detection of disease progression and deterioration and effective clinical intervention in children with SARS-CoV-2 infection.

## Materials and methods

### General information

The information on the laboratory test indices on initial admission, treatment, and prognosis of 121 children with confirmed and suspected SARS-CoV-2 infection who had been admitted to Wuhan Children’s Hospital from January 25, 2020, to February 20, 2020, was collected, compiled, and retrospectively analyzed. The indices included: peripheral blood cell counts (white blood cells, red blood cells, neutrophils, lymphocytes, eosinophils), acute infection indices (ferritin [FERR], serum amyloid A [SAA], C-reactive protein [CRP], calcitonin [PCT]), cytokines (interleukin-2 [IL-2], IL-4, IL-6, IL-10, tumor necrosis factor alpha [TNF-α], interferon gamma [IFN-γ]), and lymphocyte subsets (CD3^+^ T cells, CD3^+^CD4^+^ T helper [Th] cells, CD3^+^CD8^+^ cytotoxic T [Tc] cells, CD16^+^CD56^+^ natural killer [NK] cells, CD19^+^ B cells, CD4^+^CD25^+^ T regulatory [Treg] cells).

All children gave written informed consent signed by their legal guardians before being tested.

### Diagnostic criteria

According to the recommendations for the diagnosis, prevention, and control of COVID-19 in children (first interim edition) [5], the children were diagnosed with suspected SARS-CoV-2 infection if both of the following criteria were met:

1. Epidemiological history: History of travel or residence in Wuhan within 14 days before disease onset; or contact with patients with fever and respiratory symptoms or clusters of SARS-CoV-2 infection (in Hubei Province) within 14 days before disease onset.
2. Clinical manifestations: a) Fever, fatigue, and dry cough (some children may have no or low fever); b) possible radiological features of pneumonia such as multiple ground-glass opacities and/or infiltrative shadows in the lungs; c) normal or decreased total white blood cell count, or decreased lymphocyte count in the early stage of onset; and d) clinical manifestations that cannot be fully explained by other etiologies.

Children with suspected SARS-CoV-2 infection were confirmed if they tested positive for SARS-CoV-2 nucleic acid in nasopharyngeal or throat swabs, and lower respiratory tract secretions by real-time fluorescence RT-PCR.

The infected children can be divided into five categories [5], as follows:

1. Asymptomatic infection: The children have no clinical symptoms and signs, the chest imaging examination are normal, but the 2019⁃nCoV nucleic acid test is positive, or the serum specific antibody are positive by the retrospective diagnosis.
2. Mild: The children mainly have acute upper respiratory tract infection, including fever, fatigue, myalgia, cough, sore throat, runny nose and sneezing symptoms. Pharyngeal congestion can be seen in physical examination, but lungs have no positive signs. Some children may have no fever, but only with nausea, vomiting, abdominal pain, diarrhea or other gastrointestinal symptoms.
3. Common type: The children are manifested as pneumonia. They often have fever and cough, initially mostly dry cough, then sputum cough, and some can have wheezing but no obvious shortness of breath and other hypoxia. Coarse rales, dry rales and/or wet rales can be heard from the lungs. Some of the children did not have any clinical symptoms and signs, but chest CT showed pulmonary lesions, which were subclinical.
4. Severe type: Early respiratory symptoms such as fever and cough can be accompanied by digestive symptoms such as diarrhea. The disease usually progresses in about 1 week with dyspnea, central cyanosis, saturation of pulse oximetry < 0.92 without oxygen inhalation and other hypoxia manifestations.
5. Critical type: The children may rapidly progress to acute respiratory distress syndrome (ARDS) or respiratory failure, and may also develop multiple organ dysfunction such as shock, encephalopathy, myocardial injury or heart failure, coagulation dysfunction and acute kidney injury, which can be life-threatening.

### Disease classification

According to the above criteria, the disease was divided into mild (group I: including asymptomatic infection, and mild and common cases), severe (group II), and critical cases (group III).

### Laboratory examinations

According to the regulations of the local center for disease prevention and control, RT-PCR was used for SARS-CoV-2 nucleic acid testing [6]. RNA from the respiratory specimens was extracted with the High Pure Viral Nucleic Acid Kit (Zhongzhi, Wuhan, China). Lymphocyte subsets and cytokines were tested using a BD FACSCanto II flow cytometer (BD Biosciences). Lymphocytes were analyzed using a BD Multitest IMK Kit (cat. no. 340503, BD Biosciences); plasma cytokines (IL-2, IL-4, IL-6, IL-10, TNF-α, IFN-γ) were determined using a human Th1/2 cytokine kit II (BD Biosciences).

### Statistical analysis

Categorical variables are expressed as frequencies or percentages and were analyzed using the chi-square or Fisher exact test. Continuous variables are expressed as the mean ± standard deviation. Differences between the three groups were compared using analysis of variance. Non-parametric variables are expressed as medians and interquartile ranges (IQRs), and significance was determined using the Mann–Whitney *U* test and Kruskal–Wallis test. Correlation coefficients of continuous variables were determined using the Spearman rank correlation test. The principal clinical parameters that could be used to distinguish mild infection from severe SARS-CoV-2 infection were determined using principal component analysis (PCA). The selected parameters for mild and severe SARS-CoV-2 infection were evaluated using the receiver operating characteristic (ROC) curve and the area under the ROC curve (AUC). *P* < 0.05 was considered statistically significant. Tables 1, 2 show the specific test indices and results. All statistical analyses were conducted using SPSS Version 22.0 software (SPSS Inc), R package (v.3.6.1), and GraphPad Prism 8.0 (GraphPad Software LLC) (Tables 1, 2).

**Table 1:** Baseline Characteristics of Patients with SARS-CoV-2

Baseline variables	Overall (***N*** = 121)	Mild (***N*** = 101)	Severe (***N*** = 12)	Critical (***N*** = 8)	***P Value***
**Age (year)**					
Mean (SD)	6.25 (4.31)	6.51 (4.43)	6.36 (3.08)	2.71 (2.96)	0.054
Median [Min, Max]	6.00 [0.130, 15.0]	6.75 [0.130, 15.0]	6.96 [1.00, 10.6]	1.67 [0.170, 8.00]	
**Gender**					
Male	82 (67.8%)	65 (64.4%)	11 (91.7%)	6 (75.0%)	0.145
Female	39 (32.2%)	36 (35.6%)	1 (8.3%)	2 (25.0%)	
**PCR**					
No	36 (29.8%)	24 (23.8%)	9 (75.0%)	3 (37.5%)	0.001
Yes	85 (70.2%)	77 (76.2%)	3 (25.0%)	5 (62.5%)	
**Imaging changes**					
No	24 (19.8%)	22 (21.8%)	2 (16.7%)	0 (0%)	0.317
Yes	97 (80.2%)	79 (78.2%)	10 (83.3%)	8 (100%)	
**Dyspnoea**					
No	112 (92.6%)	98 (97.0%)	11 (91.7%)	3 (37.5%)	< 0.001
Yes	9 (7.4%)	3 (3.0%)	1 (8.3%)	5 (62.5%)	
**Cough**					
No	52 (43.0%)	43 (42.6%)	6 (50.0%)	3 (37.5%)	0.841
Yes	69 (57.0%)	58 (57.4%)	6 (50.0%)	5 (62.5%)	
**Fever**					
No	43 (35.5%)	43 (42.6%)	0 (0%)	0 (0%)	0.001
Yes	78 (64.5%)	58 (57.4%)	12 (100%)	8 (100%)	
**Gastrointestinal symptoms**					
No	108 (89.3%)	93 (92.1%)	10 (83.3%)	5 (62.5%)	0.027
Yes	13 (10.7%)	8 (7.9%)	2 (16.7%)	3 (37.5%)	
**Runny nose**					
No	110 (90.9%)	91 (90.1%)	11 (91.7%)	8 (100%)	0.641
Yes	11 (9.1%)	10 (9.9%)	1 (8.3%)	0 (0%)	
**Headache**					
No	115 (95.0%)	95 (94.1%)	12 (100%)	8 (100%)	0.535
Yes	6 (5.0%)	6 (5.9%)	0 (0%)	0 (0%)	
**Chest tightness**					
No	116 (95.9%)	96 (95.0%)	12 (100%)	8 (100%)	0.597
Yes	5 (4.1%)	5 (5.0%)	0 (0%)	0 (0%)	
**Sputum production**					
No	110 (90.9%)	91 (90.1%)	11 (91.7%)	8 (100%)	0.641
Yes	11 (9.1%)	10 (9.9%)	1 (8.3%)	0 (0%)	
**Muscle ache**					
No	117 (96.7%)	98 (97.0%)	11 (91.7%)	8 (100%)	0.533
Yes	4 (3.3%)	3 (3.0%)	1 (8.3%)	0 (0%)	
**Fatigue**					
No	112 (92.6%)	94 (93.1%)	11 (91.7%)	7 (87.5%)	0.84
Yes	9 (7.4%)	7 (6.9%)	1 (8.3%)	1 (12.5%)	
**With other diseases**					
No	113 (93.4%)	98 (97.0%)	10 (83.3%)	5 (62.5%)	< 0.001
Yes	8 (6.6%)	3 (3.0%)	2 (16.7%)	3 (37.5%)

**Table 2:** Peripheral blood indices’ Results of children with SARS-COV-2

Variables	Overall	Mild	Severe	Critical	***P Value***
**Routine Blood Result**	**(*n*** **= 120)**	**(*n*** **= 101)**	**(*n*** **= 12)**	**(*n*** **= 7)**	
Hemoglobin, Mean (SD), g/L	124 (14.6)	126.5 (13.5)	119.8 (9.1)	100.0 (13.7)	< 0.001
Platelet, Mean (SD), ×10^9^/L	260 (99.5)	264.0 (100.9)	253.3 (60.1)	210.6 (129.2)	0.381
White blood cell, Mean (SD), × 10^9^/L	7.3 (7.9)	7.7 (8.5)	6.0 (2.2)	4.0 (2.5)	0.412
Red blood cell, Mean (SD), × 10^9^/L	4.6 (0.6)	4.6 (0.5)	4.5 (0.4)	3.6 (0.5)	< 0.001
Neutrophil, Mean (SD), ×10^9^/L	3.8 (4.9)	3.9 (5.2)	4.0 (2.0)	1.8 (1.6)	0.556
Lymphocyte, Mean (SD), ×10^9^/L	2.7 (1.5)	2.9 (1.5)	1.5 (0.6)	1.7 (1.4)	0.001
Monocyte, Mean (SD), ×10^9^/L	0.7 (2.5)	0.8 (2.7)	0.5 (0.3)	0.5 (0.4)	0.909
Eosinophil, Mean (SD), ×10^9^/L	0.1 (0.2)	0.1 (0.2)	0.0 (0.0)	0.0 (0.0)	0.008
Basophil, Mean (SD), ×10^9^/L	0.0 (0.0)	0.0 (0.0)	0.0 (0.0)	0.0 (0.0)	0.373
**Immune Factor Results**	**(*n*** **= 102)**	**(*n*** **= 86)**	**(*n*** **= 9)**	**(*n*** **= 7)**	
T, Mean (SD), **/μL**	2260 (1190)	2499.0 (1113.9)	1233.1 (562.6)	607.7 (585.9)	< 0.001
Median [Min, Max]	2020 [146, 5610]	2240 [984, 5610]	1190 [543, 2180]	425 [146, 1840]	
Th, Mean (SD), **/μL**	1160 (708)	1290.6 (690.8)	590.2 (249.3)	349.4 (308.6)	< 0.001
Median [IQR]	1030 [817]	1130 [860.75]	685 [431.5]	297 [298]	
Tc, Mean (SD), **/μL**	941 (604)	1042.5 (590.3)	516.3 (317.1)	233.1 (254.1)	< 0.001
Median [IQR]	252 [307.5]	310 [306.5]	117 [100.5]	72.0 [149]	
NK, Mean (SD), **/μL**	343 (264)	382.8 (265.5)	157.3 (105.1)	91.0 (83.9)	0.001
Median [IQR]	523 [474.75]	566 [440.5]	272 [272]	254 [452.5]	
B, Mean (SD), **/μL**	644 (458)	696.0 (461.4)	324.3 (185.9)	422.0 (461.1)	0.026
Median [IQR]	1.24 [0.688]	1.24 [0.563]	1.12 [0.8]	1.86 [1.72]	
Treg, Mean (SD), **/μL**	176 (95.0)	179.8 (95.4)	120.5 (0.7)	53.0 (NA)	0.297
Median [IQR]	156 [137.25]	160 [140]	121 [1]	53.0 [NA]	
Th/Tc, Mean (SD)	1.39 (0.65)	1.3 (0.6)	1.3 (0.5)	2.0 (1.2)	0.027
Median [IQR]	4.82 [1.99]	4.81 [1.96]	4.70 [1.51]	7.16 [NA]	
**Cytokine Results**	**(*n*** **= 121)**	**(*n*** **= 101)**	**(*n*** **= 12)**	**(*n*** **= 8)**	
IL-2, Mean (SD), **pg/mL**	1.6 (0.7)	1.6 (0.7)	1.5 (0.4)	1.4 (0.3)	0.556
Median [IQR]	1.47 [0.445]	1.47 [0.44]	1.38 [0.445]	1.40 [0.61]	
IL-4, Mean (SD), **pg/mL**	3.1 (1.8)	3.1 (1.9)	3.2 (1.3)	2.7 (0.8)	0.819
Median [IQR]	2.62 [1.275]	2.54 [1.31]	2.66 [2.013]	2.76 [0.87]	
IL-6, Mean (SD), **pg/mL**	48.4 (352)	9.9 (16.2)	25.2 (24.5)	569.1 (1337.3)	< 0.001
Median [IQR]	5.30 [11.255]	4.27 [6.745]	17.7 [18.272]	73.5 [251.828]	
IL-10, Mean (SD), **pg/mL**	24.0 (145)	6.8 (8.2)	9.9 (15.8)	262.1 (536.1)	< 0.001
Median [IQR]	4.62 [3.795]	4.50 [2.87]	4.92 [3.11]	43.5 [256.065]	
TNF-α, Mean (SD), **pg/mL**	2.2 (2.7)	2.3 (3.0)	1.6 (0.5)	2.1 (1.1)	0.725
Median [IQR]	1.67 [0.815]	1.67 [0.826]	1.69 [0.743]	1.67 [1.203]	
IFN-γ, Mean (SD), **pg/mL**	31.0 (180)	8.4 (17.7)	58.8 (141.1)	274.6 (664.6)	< 0.001
Median [IQR]	3.71 [5.7]	3.45 [2.745]	13.2 [27.215]	10.8 [179.778]	
**Other Inflammatory Factor Results**	**(*n*** **= 119)**	**(*n*** **= 99)**	**(*n*** **= 12)**	**(*n*** **= 8)**	
FERR, Mean (SD), **ng/mL**	412 (1840)	88.1 (60.5)	198 (109)	3230 (5350)	< 0.001
Median [IQR]	81.8 [85.80]	68.1 [61.8]	169 [179]	984 [4406]	
Missing, no. (%)	38 (31.9%)	36 (36.4%)	2 (16.7%)	0 (0%)	
PCT, Mean (SD), **ng/mL**	1.30 (9.50)	0.217 (0.777)	0.248 (0.193)	15.3 (34.4)	< 0.001
Median [IQR]	0.060 [0.128]	0.055 [0.05]	0.185 [0.225]	2.10 [8.335]	
Missing, no. (%)	7 (5.9%)	7 (7.1%)	0 (0%)	0 (0%)	
SAA, Mean (SD), **mg/L**	51.5 (66.8)	29.1 (47.1)	121 (61.7)	198 (2.60)	< 0.001
Median [IQR]	5.00 [102.18]	5.00 [24.57]	120 [90.38]	198 [3.67]	
Missing, no. (%)	71 (59.7%)	61 (61.6%)	4 (33.3%)	6 (75.0%)	
CRP, Mean **(SD)**, **mg/L**	10.3 (16.7)	6.03 (8.13)	36.0 (32.9)	22.5 (20.0)	< 0.001
Median [IQR]	5.00 [11.5]	4.00 [7.12]	22.6 [48.48]	13.4 [22.05]	
Missing, no. (%)	4 (3.4%)	4 (4.0%)	0 (0%)	0 (0%)	

## Results

### Demographics and clinical feature analysis of the children with COVID-19

During the study period, a total of 490 children with confirmed or suspected COVID-19 were hospitalized at our hospital; among them, 121 children with complete clinical data were included in this study. The median age was 6.0 years (range: 0.13–15.0 years). There were 82 boys (67.8%) and 39 girls (32.2%). There were 101 mild (83.5%, asymptomatic infection, and mild and common cases), 12 severe (9.9%), and 8 critical (6.6%) cases.

Upon admission, the children’s symptoms mainly included fever (78 cases, 64.5%) and cough (69 cases, 57.0%); minor symptoms included gastrointestinal symptoms (13 cases, 10.7%), nasal discharge (11 cases, 9.1%), sputum (11 cases, 9.1%), fatigue (9 cases, 7.4%), respiratory distress (9 cases, 7.4%), headache (6 cases, 5.0%), chest distress (5 cases, 4.1%), and muscle soreness (4 cases, 3.3%). Eighty-five patients (70.2%) tested positive for SARS-CoV-2 nucleic acid on admission; 97 patients (80.2%) had radiological changes in the lungs, with typical radiological changes of ground-glass opacities in both lungs; 85 children (70.2%) were confirmed cases, and 36 children (29.8%) were suspected cases.

The clinical symptoms of the children in groups I and II were relieved after treatment, and all were discharged after meeting the criteria of cure. The median lengths of hospital stay of the children in groups I and II were 11.0 days (IQR: 9.0–14.0 days) and 10.0 days (IQR: 8.75–12.5 days), respectively. The length of hospital stay between the two groups was not significantly different (*P* = 0.9). As of March 16, 2020, 2 patients in group III were still hospitalized, and 1 patient in group III died 36 days after admission, with a median hospital stay of 20.5 days (IQR: 17.25–35.25 days). There was no progression from mild to severe or from severe to critical cases among the hospitalized children.

### Analysis of the peripheral blood indices of the children infected with SARS-CoV-2

**Fig. 1:**
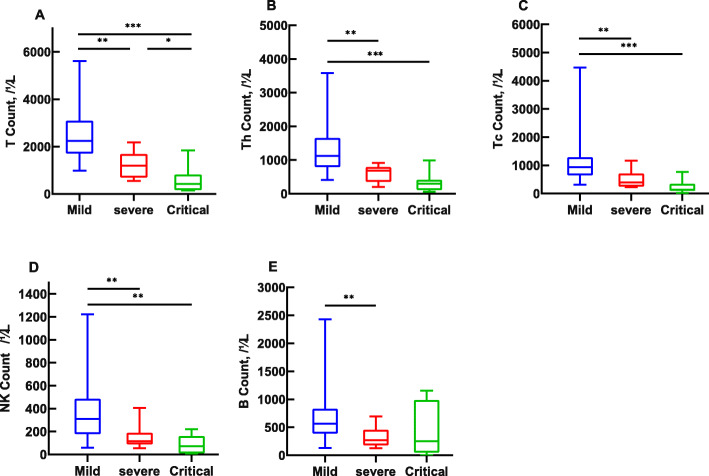
Peripheral Blood Lymphocyte Subset Counts and T Cell–derived Cytokine Concentrations on Admission in Children with COVID-19. Peripheral blood lymphocyte subset counts [CD3^+^CD4^+^ T cells (Th), CD3^+^CD8^+^ T cells (Tc), CD16^+^CD56^+^ NK cells (NK), CD19^+^ B cells (B)] in the common (group I), severe (group II), and critical (group III) cases were analyzed after hospital admission. Media [min, max]; *, p < 0.05; **, p < 0.01, ***, p < 0.001. Results were tested for significance with the Kruskal–Wallis test

The Kruskal–Wallis test and Spearman correlation analysis of the initial laboratory examinations on admission (Table 2, Fig. 1) of the 121 children showed that there was a significant decrease from groups I to III in CD3^+^CD4^+^ Th cell (*P* < 0.001), CD3^+^CD8^+^ Tc cell (*P* < 0.001), and CD16^+^CD56^+^ NK cell (*P* = 0.001) counts. Groups II and III had significantly lower CD19^+^ B cell counts than group I (*P* = 0.026). Group III had a significantly higher Th/Tc cell ratio than groups I and II (*P* = 0.027).

**Fig. 2:**
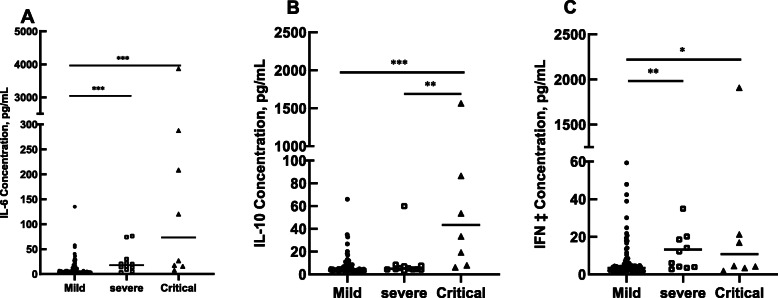
T Cell–derived Cytokine Concentrations on Admission in Children with COVID-19 on Admission in Children with COVID-19. T cell–derived cytokine concentrations [**a** interleukin-6 (IL-6), **b** IL-10, **c** interferon-γ (IFN-γ)] in the common (group I), severe (group II), and critical (group III) cases were analyzed after hospital admission. Media [min, max]; *, p < 0.05; **, p < 0.01, ***, p < 0.001. Results were tested for significance with the Kruskal–Wallis test

**Fig. 3:**
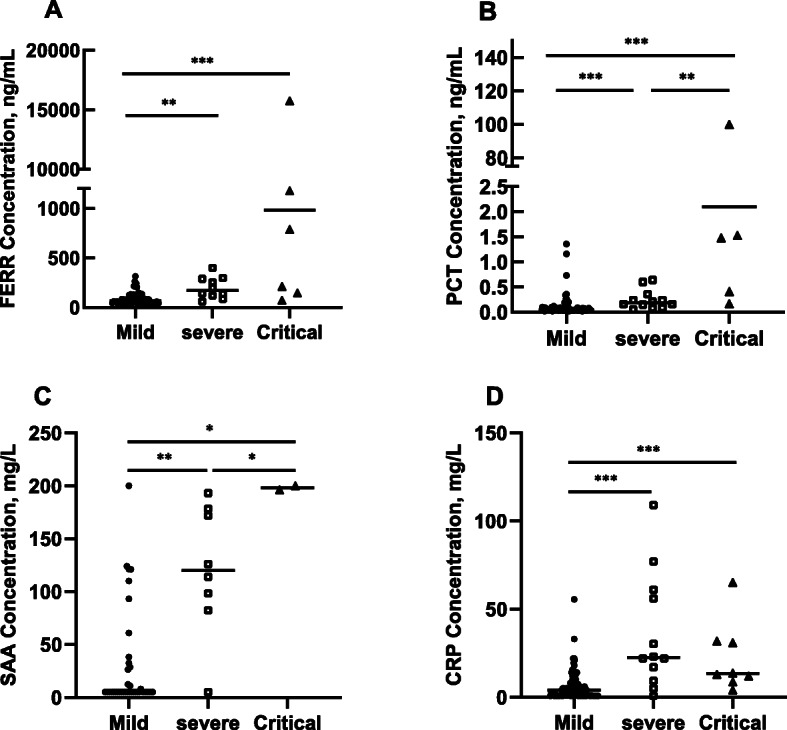
Analysis of Inflammatory Factor Concentrations on Admission in Children with COVID-19. **a** Ferritin (FERR), **b** calcitonin (PCT), **c** serum amyloid A protein (SAA) and **d** C-reactive protein (CRP) concentrations in the peripheral blood from common (group I), severe (group II), and critical (group III) cases were analyzed after hospital admission. Median [min, max]; *, p < 0.05; **, p < 0.01, ***, p < 0.001. Results were tested for significance with the Kruskal–Wallis test

From group I to III, there was a significant increase in IL-6, IL-10, and IFN-γ (Table 2, Fig. 2), and FERR, SAA, and PCT (Table 2, Fig. 3), i.e., with increasing disease severity (*P* < 0.001). Although CRP was significantly higher in groups II and III than in group I (*P* < 0.001), its highest mean value was highest (36.0 ± 32.9 mg/L) in group II.

The results of routine blood testing on admission showed a decrease from group I to III for hemoglobin (*P* < 0.001), red blood cell count (*P* < 0.001), and eosinophil count (*P* = 0.008). Groups II and III had significantly lower lymphocyte counts than group I (*P* = 0.001), with group II having the lowest count. Other routine blood parameters between the three groups were not significantly different (Table 2).

It should be noted that group III was younger, potentially including very young infants, even though the ANOVA testing did not reach statistical significance across groups (Table 3).

**Table 3:** Demographic and Clinical Features of Critical Patients

	Critical 1	Critical 2	Critical 3	Critical 4	Critical 5	Critical 6	Critical 7	Critical 8	Aggregate
Sex	Male	Male	Female	Male	Male	Male	Male	Female	6/8 Male
Age (years)	6.50	0.17	0.21	2.58	1.08	2.25	8.00	0.87	Median 1.67 years old
Ventilator	No	No	Yes	No	Yes	No	Yes	Yes	4/8
PCR	Pos	Pos	Neg	Neg	Neg	Pos	Pos	Pos	5/8
Imaging changes	Yes	Yes	Yes	Yes	Yes	Yes	Yes	Yes	8/8
Dyspnoea	No	Yes	Yes	No	Yes	Yes	No	Yes	5/8
Cough	Yes	Yes	Yes	No	No	Yes	Yes	No	5/8
Fever	Yes	Yes	Yes	Yes	Yes	Yes	Yes	Yes	8/8
Gastrointestinal symptoms	Yes	No	Yes	No	No	No	No	Yes	3/8
**Comorbidities**									
Acute lymphoblastic leukemia	Yes	No	No	No	No	No	Yes	No	2/8
Intussusception	No	No	No	No	No	No	No	Yes	1/8
**Complications**									
Hemophagocytic lymphohistiocytosis	No	No	No	Yes	No	No	No	No	1/8
Spesis	No	No	No	No	Yes	No	Yes	Yes	3/8
Non-infectious multiple organ ysfunction syndrome (MODS)	No	No	No	No	Yes	No	No	Yes	1/8
Cardiac dysfunction	No	No	No	Yes	Yes	No	Yes	No	3/8
Renal dysfunction	No	Yes	No	Yes	Yes	No	No	No	3/8
Abnormal liver function	No	Yes	No	Yes	No	No	No	No	2/8
Coagulation disorders	No	No	No	Yes	No	No	No	Yes	2/8
Epilepsy	No	No	No	No	No	No	No	Yes	1/8

Demographic and clinical features of MIS-C patients (*n* = 8)

### Prognostic factors for early identification of critical COVID-19

**Fig. 4:**
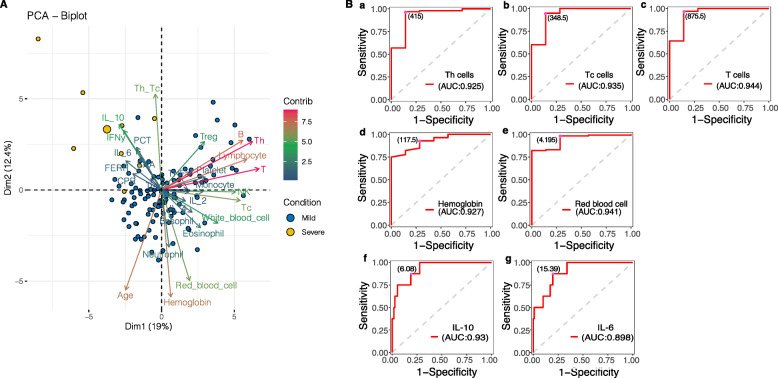
Prognostic Factors of Children with Critical COVID-19. **a** PCA was used to identify correlated variables for distinguishing critical cases (group B) from common and severe cases (group A) on admission. The 10 most contributory variables, i.e., CD3^+^ T cells (T), red blood cells, CD3^+^CD8^+^ T cells (Tc), interleukin-10 (IL-10), hemoglobin, CD3^+^CD4^+^ T cells (Th), IL-6, lymphocytes, CD19+ B cells (B), and interferon-γ (IFN-γ) were identified. **b** The ROC curve and AUC were calculated for the 10 parameters using the R package pROC. T, Th, Tc, IL-6, IL-10, red blood cells, and hemoglobin had AUC values of > 0.85

Next, we explored the possibility of early identification of children with critical COVID-19 using the above peripheral blood parameters as prognostic factors. PCA using the R package factoextra revealed a clear difference between the three groups, with more significant differences between groups I + II and group III (Fig. 4a). Therefore, groups I and II were combined into group A, and group III was designated group B for PCA and comparisons (Fig. 4b).

A comprehensive analysis of the contributory values suggested that 10 indices (red blood cells [RBC], hemoglobin [Hb], lymphocytes [Lym], T lymphocytes [T], Th cells [Th], Tc cells [Tc], B lymphocytes [B], IL-6, IL-10, IFN-γ) can be used as potential prognostic factors, and further statistical analysis was performed on these indices.

To determine the diagnostic value of the above 10 indices, we calculated the AUC of the ROC curve. The AUC values of T (0.944), RBC (0.941), Tc (0.935), IL-10 (0.93), Hb (0.927), Th (0.925), and IL-6 (0.898) were greater than that of Lym (0.718), B (0.708), and IFN-γ (0.688). Meanwhile, the cutoff values for T (875.5), RBC (4.195), Tc (348.5), IL-10 (6.08), Hb (117.5), Th (415.0), IL-6 (15.39), Lym (1.77), B (342.0), and IFN-γ (4.24) were calculated according to the Youden index of the ROC curve. T, Th, Tc, IL-6, IL-10, RBC, and Hb had AUC values of > 0.85, indicating that they contributed significantly to the identification of critical cases in this study.

## Discussion

The diagnosis and treatment of severe or critical in children with COVID-19, especially infants and young children, are very different from that of adults. Symptom-based clinical evaluations of children are limited by their inability to participate in examinations, essentially rendering the process of diagnosis and treatment very challenging. Therefore, determining the disease condition with the aid of laboratory tests in the early stage of SARS-CoV-2 infection in children with unspecific symptoms is very important. Children are susceptible to SARS-CoV-2; unlike adult patients, most child patients present with mild symptoms, recover quickly, and have a relatively good prognosis [1, 7]. As of February 20, 2020, a total of 12 children with severe COVID-19 and 8 children with critical COVID-19 had been admitted to our hospital, significantly fewer than the severe and critical adult cases [7–9].

In adults infected with SARS-CoV-2, the loss of T cells may lead to increased inflammatory response, while the recovery of T cell numbers helps to alleviate the degree of inflammatory response [10, 11]. Here, we show that, in children infected with SARS-CoV-2, the laboratory examination indices of the peripheral blood in the early stage of infection had similar trends to that of adults. Patients with severe and critical COVID-19 had significantly reduced T lymphocyte numbers (especially CD8^+^ T lymphocytes) and increased Th/Tc ratios [12–15]. Children with severe disease had significantly increased inflammation-related indices (FERR, SAA, PCT) and cytokines (IL-6, IL-10, IFN-γ), which was contrary to the trend for T cells. This suggests that the immune systems, especially the immune cells, of children in the growth and development stage are quickly activated to resist the invasion and damage of SARS-CoV-2 infection. Existing reports show that adults infected with SARS-CoV-2 seemed to have inferior immune response compared to children in our study (lymphocyte: (0.6–0.9) × 10^9^/L in adults vs. 1.7 × 10^9^/L in children), and adults with severe disease had more pronounced immune cell failure on initial admission [7–9, 11]. This may partly explain the lower proportions of severe and critical cases of SARS-CoV-2 infection in children than in adults, especially compared to the elderly [1, 7]. However, the limited data in the present study necessitates further study of the mechanism underlying the difference between children and adults.

In the early stage of infection, influenza viruses induce IL-6 production in the epithelial and endothelial cells of the lungs, which recruits and activates CD8^+^ T lymphocytes and NK, Treg, and Th2 cells to secrete IFN-γ, IL-10, and IL-5 to eliminate the viruses and virus-infected cells, inhibit the exacerbation of inflammation, and restore lung function [16]. However, a sharp increase in IL-6 may be associated with poor clinical prognosis [17–20]. High IL-10 levels suggest that the body attempts to regulate the inflammatory damage caused by other actors in the cytokine storm, which indicates, to a large extent, that the inflammatory state may already be uncontrollable [21–25]. IFN-γ has potent antiviral activity, and NK cells produce IFN-γ in the early stage of viral infection (3–5 days after infection) to inhibit viral replication [26]. Carl A. Pierce et.al [27] reported that The neutralizing antibody concentration was higher in infected adults than in children, and the neutralizing antibody titer was inversely correlated with the serum IL-17A and IFN-γ concentrations. In children, early immune responses mediated by cells that produce IL-17A and IFN-γ lead to more rapid resolution of viral infection and may have mitigated against the progressive cytokine release and tissue pathology that occurs with more robust adaptive immune responses, which means the boosted early innate immune responses may be important.

Here, the IL-6, IL-10, and IFN-γ initially tested on admission increased from group I to group III, with groups II and III having significantly higher values than group I (*P* < 0.001). This suggests that, in the children with severe and critical disease, IL-6, IL-10, and IFN-γ had been produced to resist viral invasion and there had been attempts to suppress the inflammatory response in the early stage of the SARS-CoV-2 infection.

The three cytokines were significantly higher in group III than in groups I and II, but group III had significantly lower Th and Tc cell counts than groups I and II, indicating that relatively severe inflammatory damage had occurred in the early stage of SARS-CoV-2 infection. The acute inflammatory indices, i.e., FERR, SAA, and PCT, were also significantly higher in group III than in groups I and II, indicating that the disease conditions of the children in group III had deteriorated rapidly, their immune system had become dysregulated, and cytokine storm was inevitable.

We found that the children in group II had relatively good treatment outcomes and similar prognoses to the children in group I, and no case progressed from group II to group III, which suggests that the pathogenesis of critical COVID-19 in children warrants further research.

We further classified the children with critical disease as group B and combined groups I and II into group A. PCA and ROC curve analysis of the two groups revealed that the indices T (0.944), RBC (0.941), Tc (0.935), IL-10 (0.93), Hb (0.927), Th (0.925), and IL-6 (0.898) had relatively larger AUC values, with cutoff values of 875.5, 4.195, 348.5, 6.08, 117.5, 415.0, and 15.39, respectively. This suggests that the seven indices are of great contribution to the early identification and prediction of the prognosis of children with critical COVID-19.

Mild patients in this study did not have typical multisystem inflammatory syndrome in children (MIS-C) phenotypes. Only one critical child appeared to have MIS-C phenotypes, according to Gruber CN PR, Camila Rosat Consiglio et.al, [28, 29] during the treatment process. Follow-up 4 months later was showed that peripheral blood indexes were almost normal, but abnormal liver, kidney, myocardium and coagulation function remained.

In short, as children are susceptible to SARS-CoV-2, it is particularly important to preliminarily distinguish between mild and severe disease through simple testing at the initial stage of admission. Our study suggests that seven indices (T, Th, Tc, IL-6, IL-10, RBC, Hb) are helpful for early diagnosis of COVID-19 severity in children, and may provide a laboratory basis for clinicians to offer timely treatment to children with critical COVID-19. Due to the limited sample size, further multi-center, large-sample studies are needed.

## Data Availability

The datasets generated during and/or analyzed during the current study are available from the corresponding author on reasonable request. Patients have not been reported in any other submission by anyone.
